# Pilot study: *In vitro* reduction of hemoglobin from canine blood with hemoperfusion using the Cytosorb^®^ adsorber

**DOI:** 10.1371/journal.pone.0328306

**Published:** 2025-07-28

**Authors:** Kathrin Spiegel, René Dörfelt, Katrin Hartmann, Florian Sänger

**Affiliations:** LMU Small Animal Clinic, Centre for Clinical Veterinary Medicine, Faculty of Veterinary Medicine, LMU München, Munich, Germany; Hamadan University of Medical Sciences, IRAN, ISLAMIC REPUBLIC OF

## Abstract

Cell-free hemoglobin (cfHb) can be toxic and lead to kidney injury. This study assessed the *in vitro* reduction of cfHb from canine hemolyzed blood using hemoperfusion with a Cytosorb® cytokine adsorber. Canine whole blood was processed in linear and circular setups, with three runs each, at 100 mL/min. Hemolysis and osmolarity adjustments were performed with distilled water and hypertonic saline. Anticoagulation was optimized with heparin (10,000 IU/L). A median of 3.38 L of hemoglobin solution was processed in the linear setup. Samples were collected after the adsorber and from the waste bag. In the circular setup, a median of 2.09 L was processed, with samples taken before and after the adsorber. CfHb concentration was measured using the XT-2000iV® hematology analyzer (Sysmex). A control setup without an adsorber was run for 24 hours to assess cfHb stability. In the linear setup, cfHb concentration decreased by a median of 17.8% (14.7–26.8%), from 1.7 mmol/L to a minimum of 0.9 mmol/L, with 12.1 g (11.9–23.5 g) of cfHb removed. The median cfHb concentration after the adsorber displayed a logarithmic increase from 0.9 mmol/L (0.8–1.2 mmol/L) to 1.6 mmol/L (1.4–2.1 mmol/L). After processing 2.4 L, no further reduction occurred. In the circular setup, cfHb was reduced by a median of 41.3% (46.1–45.0%), representing 17.4 g (14.6–19.0 g) removed after 13.0 L (13.0–14.0 L). The reduction plateau was reached after 13 L. The hemoglobin reduction ratio in the circular setup at 3 L processed cfHb-solution was 25.0% (23.1–33.3%) and was not different from the linear setup (p = 0.400). The cfHb clearance decreased in both setups over time. CfHb concentration in the control setup was stable for 24 hours. Hemoperfusion with the Cytosorb^®^ adsorber reduced cfHb *in vitro* from a canine blood solution.

## Introduction

Diseases associated with severe hemolysis, such as babesiosis or immune-mediated hemolytic anemia, can lead to fatal hemolysis in humans and animals. Physiologically, cell-free hemoglobin (cfHb) from hemolysis is bound by different binding agents, such as haptoglobin. However, in cases of severe intravascular hemolysis, the binding capacity is exhausted, and cfHb circulates freely in the vascular system, becoming toxic to the organism in various ways [1–4]. CfHb exerts detrimental effects on different physiological processes. It contributes to myocardial injuries, endothelial barrier disruption, and oxidative DNA damage. In guinea pigs with endotoxemia in combination with lipopolysaccharide, enhanced phagocyte activation, myocardial iron deposition, and heme-oxygenase-1 expression were demonstrated as a consequence of hemolysis [5,6]. Uncontrolled heme-oxygenase-1 expression leads to increased iron deposition, which in turn exacerbates oxidative stress and immunosuppression. Conversely, enhanced expression of heme-oxygenase-1 can lead to accelerated destruction of erythrocytes [7,8]. At the same time, cfHb depletes intracellular ascorbate and reduces nitrite to nitric oxide (NO), resulting in vasodilation, increased blood flow, platelet activation and end-organ injury [9]. Oxygen-related toxicity of cfHb leads to elevated oxygen partial pressure in the terminal vessels, triggering vasoconstriction, hypertension, and ischemia [4,10]. These oxidative effects can result in organ failure, particularly acute kidney injury, due to proximal tubule destruction. Hemoglobin deposits in the kidneys can also lead to tubular barrier destruction, causing acute kidney injury [11–14]. High levels of cfHb (up to 38.76 mmol/L) can cause enhanced vascular permeability, promoting non-cardiogenic pulmonary edema and acute respiratory distress syndrome (ARDS) [13,15]. Elevated cfHb concentrations are also associated with higher mortality rates in sepsis, systemic and pulmonary inflammation, and endothelial injury [13,16].

Blood purification techniques, such as hemodialysis, plasmapheresis, and hemoperfusion, have been developed to eliminate endogenous and exogenous substances. Some of these techniques are increasingly applied to veterinary patients [17,18]. Most of these extracorporeal blood purification techniques are insufficient to remove cfHb. Hemodialysis primarily targets smaller molecules, such as urea and creatinine. High-molecular-weight substances, such as hemoglobin, are too large to pass through the dialysis membrane. The molecular weight decreases the efficacy of conventional dialyzers to eliminate cell-free hemoglobin from circulation. Also, high-flux dialyzers, which process larger molecules, are still unsuitable for cfHb elimination [19].

During plasmapheresis, plasma is separated from blood cells and replaced with donor plasma and other substitute solutions, such as synthetic colloids and albumin. It aims to eliminate protein-bound toxins from the circulation but requires large amounts of donor plasma. Plasmapheresis has limited efficacy for removing cfHb from the circulation, primarily due to the non-selective nature of the procedure [20]. Plasmapheresis typically removes around 60–70% of substances within the plasma during a single plasma exchange session, with an exchange of 1.5 times the plasma volume. However, this efficiency diminishes with each additional plasma volume treated, which is insufficient for significantly reducing cfHb levels, especially in severe hemolysis where cfHb is present in high concentrations. Moreover, during plasmapheresis, a dilution of plasma proteins and coagulation factors with replacement fluids occurs, which makes the complete removal of cfHb even more challenging [21,22].

Hemoperfusion utilizes an extracorporeal circuit with an adsorber, designed for toxin elimination [23]. One recent adsorber is the Cytosorb^®^ adsorber, which was developed to eliminate inflammatory mediators and other toxins from the circulation. It has received emergency use authorization (EUA) from the FDA in the United States for cytokine removal [24], and anticoagulants (Ticagrelor and Rivaroxaban) elimination for emergent cardiopulmonary bypass surgery [25–27]. The Cytosorb^®^ adsorber has been approved in the European Union since 2011 [28]. The device is a whole blood adsorber containing polystyrene-divinylbenzene polymer beads, providing a surface area of over 45,000 square meters. A concentration gradient of the adsorbed substance and biomechanical mechanisms promote adsorption and ensure that a physiological amount of endogenous molecules remain in the circulation [29–31]. The Cytosorb^®^ adsorber primarily binds hydrophobic molecules with a molecular weight of 5–60 kilodaltons (kDa), encompassing both endogenous substances, such as bilirubin, myoglobin, or cfHb, and exogenous substances, such as drugs or toxins (e.g., aflatoxin) [18,32–38]. Its efficacy in removing cfHb has been demonstrated in humans during complex cardiac surgery and in critically ill patients [1,39]. It was also used in humans with severe sepsis, [40,41] systemic inflammation due to COVID-19 [42,43], and cardiovascular surgery to clear the blood of cfHb and inflammatory mediators, preventing post-surgical organ dysfunction [25]. A case report highlighted the reduction of cfHb with the Cytosorb^®^ adsorber during extracorporeal life support (ECLS) in humans within 48 and 87 hours, using multiple adsorbers [44].

Data concerning the application of the Cytosorb^®^ adsorber in veterinary medicine are limited. The VetResQ^®^ adsorber, a veterinary brand of the Cytosorb^®^ adsorber, combined with therapeutic plasma exchange, was successfully used to treat immune-mediated hemolytic anemia in three dogs [45]. A case series of five dogs demonstrated the efficient reduction of serum bilirubin using the Cytosorb^®^ adsorber, surpassing the effect of hemodialysis alone [46]. However, the adsorption capacity of the Cytosorb^®^ adsorber on cfHb remains unknown.

This pilot study aimed to evaluate the *in vitro* adsorption capacity of cfHb from canine hemolyzed blood with the Cytosorb^®^ adsorber during hemoperfusion.

## Materials and methods

### Ethical approval

This study was approved by the ethics committee of the Centre of Clinical Veterinary Medicine of the LMU Munich (approval no. AZ 335-19-10-2022).

### Study design

Expired canine blood from the in-house veterinary blood bank was used to prepare the cfHb solutions. This decision was made based on ethical and resource management considerations, not to withhold blood from patients urgently requiring blood transfusions. Expired blood was still sufficient for the *in vitro* experimental setup, as only hemoglobin, but not coagulation factors or intact red blood cells, were required. Blood was collected and processed in accordance to the in-house blood banking standards. A commercial blood bag (Compoflex^®^ CPDA blood bag, Fresenius Kabi AG, Bad Homburg, Germany) with an attached 16-gauge needle was used to collect a maximum of 12 mL/Kg of blood, gravity-driven from the jugular vein of the donor dogs.

The expired canine whole blood, which was stored with Citrate-Phosphate-Dextrose-Adenine at −30°C, was thawed and mixed with distilled water (aqua ad iniectabilia, B. Braun Vet Care AG, Melsungen, Germany) at a 1:3 ratio to achieve complete hemolysis, which was confirmed through microscopic evaluation before initiating the procedure. To normalize the osmolarity of the cfHb solution, 7.5% hypertonic saline (hypertonic NaCl 7.5 g/100 mL, B. Braun Vet Care AG, Melsungen, Germany) was added after hemolysis. Anticoagulation was achieved with 10,000 IU/L of heparin (Heparin-Natrium 25,000 I.E./5 mL Inj. Lsg., B. Braun Vet Care AG, Melsungen, Germany). The cfHb solution was prepared before each test run, resulting in different starting concentrations of cfHb. The resulting cfHb solution was stored in a 5-liter bag (Haemotronic S.p.A., Modena, Italy). CfHb content was analyzed using the XT-2000iV^®^ blood analysis device (Sysmex Deutschland GmbH, Norderstedt, Germany).

Hemoperfusion was performed using the blood purification platform Pure Adjust^®^ (Nikkiso Europe GmbH, Langenhagen, Germany) with a blood tubing line for apheresis^®^ (Nikkiso Europe GmbH, Langenhagen, Germany) and the Cytosorb^®^ adsorber (CytoSorbents Inc., New Jersey, United States of America). The extracorporeal circuit was primed with 200 mL of isotonic saline (NaCl 0.9%, B. Braun Vet Care AG, Melsungen, Germany).

Two distinct experimental setups, linear and circular, were applied. Each of them was conducted three times. Additionally, one run with the circular setup but without the Cytosorb^®^ adsorber was performed to prove the stability of the cfHb concentration.

**Fig 1:**
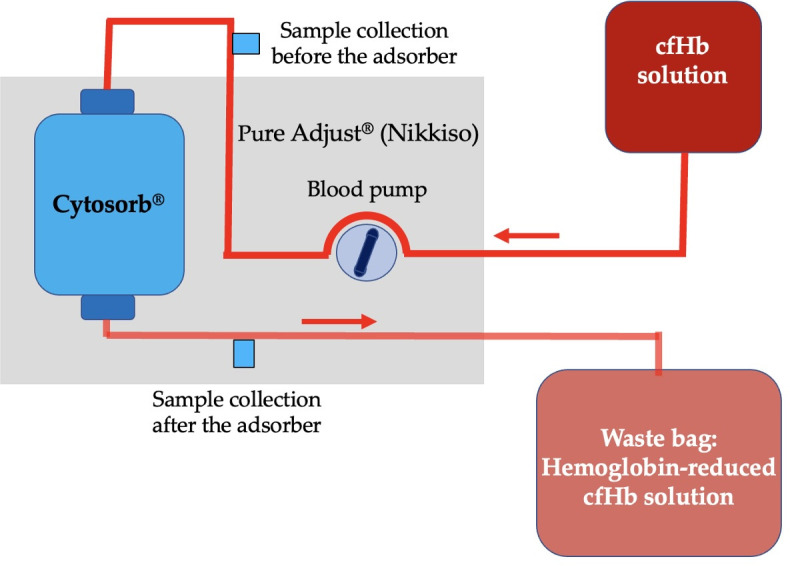
Illustration of the linear setup of a canine cell-free hemoglobin (cfHb) solution processed by hemoperfusion using the Cytosorb^®^ adsorber. The cfHb solution is passed through the Cytosorb^®^ adsorber of the Pure Adjust^®^ (Nikkiso) blood purification platform and collected in a waste bag at a blood flow rate of 100 mL/min. Samples are collected at the sample collection port after the adsorber.

The linear setup was the basic setup of the experiment. The aim was to determine the adsorption capacity of the cfHb with the Cytosorb^®^ adsorber until a stable cfHb concentration was achieved. The cfHb solution underwent continuous processing through the adsorber with a flow rate of 100 mL/min in the linear setup (Fig 1). This setup mimics a patient with a stable cfHb concentration due to a large blood volume and/or ongoing hemolysis. Samples were collected after the adsorber every 0.1 L of processed cfHb solution volume for the first liter, every 0.2 L from one liter to 1.6 L, and every 0.4 L from 1.6 L of processed blood volume.

**Fig 2:**
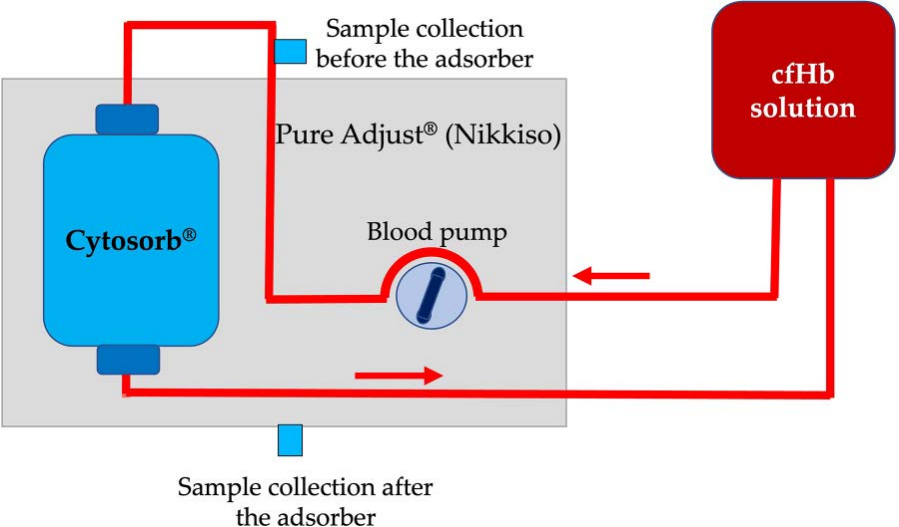
Illustration of the circular setup of a canine cell-free hemoglobin (cfHb) solution processed by hemoperfusion using the Cytosorb^®^ adsorber. The cfHb solution is processed by the Pure Adjust^®^ (Nikkiso) blood purification platform and the Cytosorb^®^ adsorber and recirculated into the same storage bag, with a flow of 100 mL/min. Samples were collected at the collection ports before and after the adsorber.

The circular setup involved the recirculation of the cfHb solution into the storage bag, using a blood flow of 100 mL/min. It was designed to mimic hemoperfusion in a patient with a small blood volume and no ongoing hemolysis (Fig 2). Samples were collected every 0.2 liter, before and after the adsorber, during the first liter passing through the adsorber, and at 1.5 and 2 L. From 3 L on, samples were collected for every liter of processed cfHb solution. This setup was run until the cfHb concentration in the storage bag was stable for 10 L of processed cfHb solution.

The starting point for cfHb analysis during the procedure in both setups was defined as 0 when 0.2 liters of Hb solution filled the system, ensuring adequate cfHb concentration at collection ports, as the Cytosorb^®^ adsorber contains a priming volume of 0.15 L and the tubing system 0.05 L of isotonic saline.

**Fig 3:**
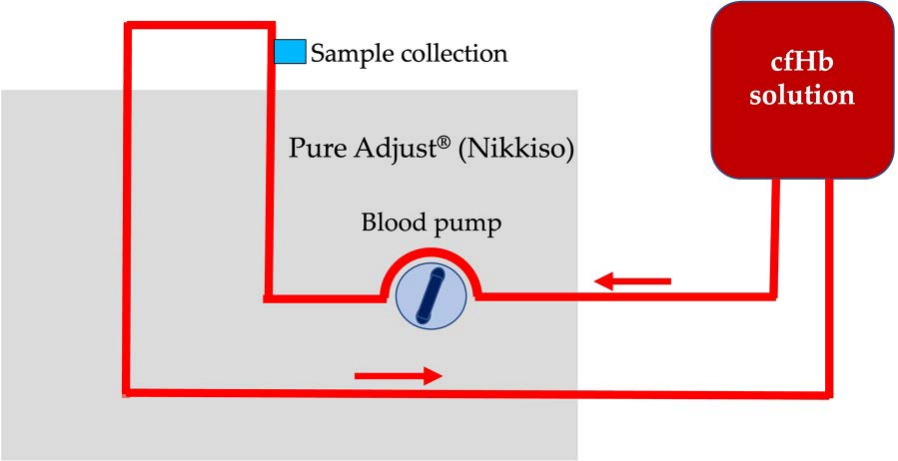
Illustration of the control setup: continuous circulation of canine cell-free hemoglobin (cfHb) solution without the Cytosorb^®^ adsorber was performed. The cfHb solution was processed using the Pure Adjust^®^ (Nikkiso) blood purification platform and recirculated into the same storage bag with a flow rate of 100 mL/min for 24 hours.

Additionally, a control setup, identical to the circular setup, but without the Cytosorb^®^ adsorber, was run for 24 hours to exclude spontaneous degradation of cfHb (Fig 3). Samples were collected after 0.5 L, 1 liter, 5 L, 10 L, 20 L, 45 L, and 144 L of processed cfHb solution.

### Statistical analysis

A commercial software (Microsoft Excel^®^, Microsoft^®^ Corporation, Redmond, USA) was used for data documentation. Statistical analysis was performed using commercially available software (Prism 5 for Windows^®^; GraphPad Software, San Diego, CA). Data are presented as median and range (minimum–maximum). Clearance was calculated with the following formula: Clearance (mL/min) = flow (mL/min) x extraction coefficient. CfHb reduction ratio was compared using the Mann-Whitney U-test. The decrease and increase of the cfHb concentrations pre- and post-absorber were compared using the Friedman test with post-hoc Dunn´s multiple comparison test. P-values < 0.05 were considered significant.

## Results

### Linear setup

**Fig 4:**
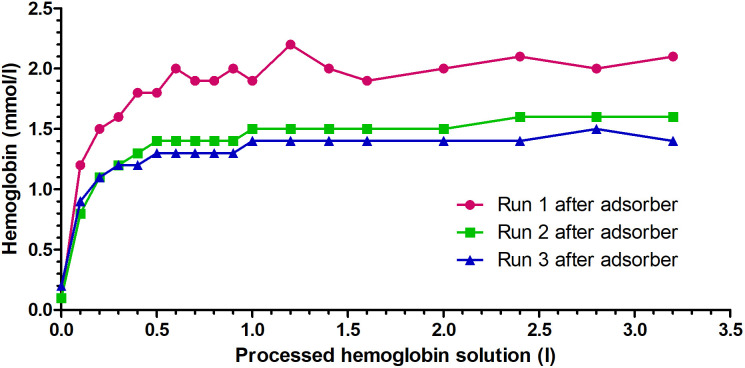
Hemoglobin concentration of three canine cell-free hemoglobin (cfHb) solutions undergoing hemoperfusion using the Cytosorb^®^ adsorber with the linear setup, analyzed after the adsorber.

**Fig 5:**
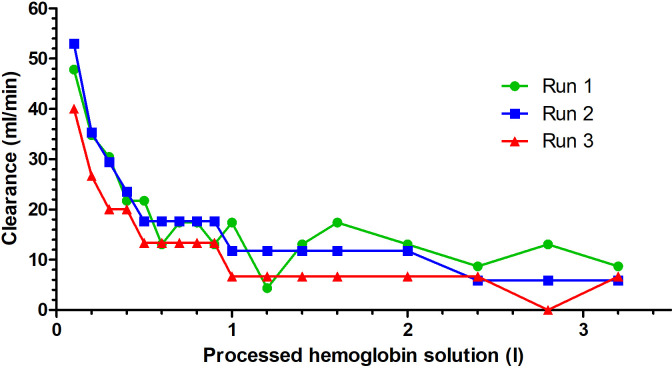
Hemoglobin clearance of three canine cell-free hemoglobin (cfHb) solutions undergoing hemoperfusion using the Cytosorb^®^ adsorber with the linear setup.

The linear setup processed a median of 3.3 L (3.0–3.4 L) of cfHb solution. After calculating the dilutional effect of the priming solution, the initial cfHb concentration in the canine cfHb solution was reduced by a median of 17.8% (range 14.7 − 26.8%). A median cfHb reduction of 12.1 g (11.9–23.5 g) was achieved during the three runs (Table 1). The median cfHb concentration after the adsorber displayed a logarithmic increase from 0.9 mmol/L (0.8–1.2 mmol/L) to 1.6 mmol/L (1.4–2.1 mmol/L) up to a median blood volume of 2.4 liters of processed cfHb solution (Fig 4). After these 2.4 liters, no further reduction of the cfHb content was observed. The cfHb concentration differed significantly between the time points (p < 0.001). Due to the low number of samples, no specific time point for the cfHb difference was found during the post-hoc analysis. The cfHb clearance decreased from 47.8 ml/min (40.0–52.9 ml/min) at 0.1 liters processed cfHb-solution to 6.7 ml/min (5.9–8.7 ml/min) at 3.2 liters processed cfHb solution (Fig 5).

**Table 1:** cfHb volume, hemoglobin concentration, and hemoglobin reduction of canine cell-free hemoglobin (cfHb) solution undergoing hemoperfusion, applying the Cytosorb^®^ adsorber in the linear and the circular setup and control run without the adsorber. The end hemoglobin concentration refers to the hemoglobin concentration in the waste bag after processing the whole cfHb solution. The dilutional effect of saline used for priming was already addressed during the calculation of the hemoglobin reduction.

Test run	Volume cfHb solution (L)	Starting hemoglobin concentration (mmol/L)	End hemoglobin concentration (mmol/L)	Hemoglobin reduction (%)	Total hemoglobin reduction (g)
**Linear setup**					
1	3.0	2.3	1.7	26.8	23.5
2	3.4	1.7	1.4	14.7	11.9
3	3.3	1.5	1.2	17.8	12..1
**Circular setup**					
1	2.1	1.3	0.7	36.1	14.6
2	2.0	1.2	0.6	45.0	17.4
3	2.2	1.2	0.7	41.3	19.0
**Control setup**					
1	2.1	0.6	0.6	0	0

### Circular setup

**Fig 6:**
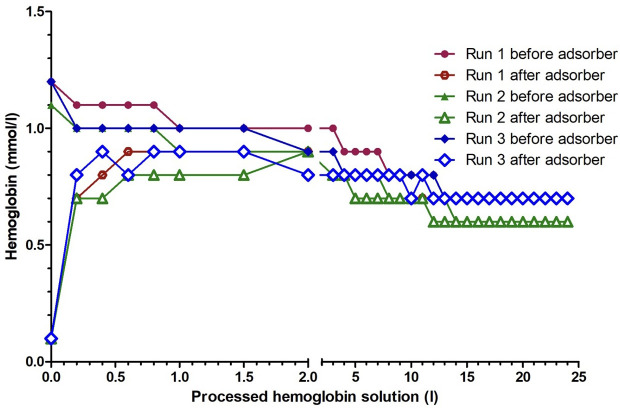
Hemoglobin concentration of three canine cell-free hemoglobin (cfHb) solutions undergoing hemoperfusion using the Cytosorb^®^ adsorber with the circular setup, analyzed before and after the adsorber. In the circular setup, samples were taken more frequently for the first two L of processed cfHb solution. The scale on the x-axis is presented in different intervals, as most changes occurred in the first two L of processed cfHb solution.

**Fig 7:**
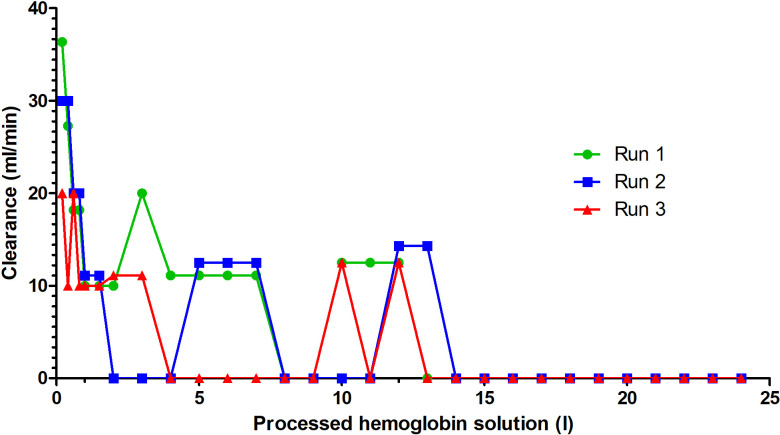
Hemoglobin clearance of three canine cell-free hemoglobin (cfHb) solutions undergoing hemoperfusion using the Cytosorb^®^ adsorber with the circular setup.

A median of 2.1 L (2.0–2.2 L) of the canine cfHb solution was processed during the circular test runs. The cfHb concentration was reduced by a median of 41.3% (46.1–45.0%). The initial median cfHb concentration of the canine cfHb solution, of 1.2 mmol/L (1.2–1.3 mmol/L) before the first run, steadily decreased before and increased after passing through the Cytosorb^®^ adsorber. This continuous reduction resulted in a minimal cfHb concentration of 0.7 mmol/L (0.6–0.7 mmol/L). This represented an absolute cfHb reduction after calculating the dilutional effect of the priming saline of 17.4 g (14.6–19.0 g; Table 1). This reduction was achieved after a median of 13.0 L (13.0–14.0 L) of processed cfHb solution (Fig 6). Thereafter, no further decrease in cfHb concentration was observed. The hemoglobin reduction ratio at 3 L processed cfHb-solution was 25.0% (23.1–33.3%) and did not differ from the linear setup (p = 0.400). The pre-adsorber cfHb concentration decreased, and the post-adsorber cfHb concentration increased significantly between the time points (p < 0.001). Due to the low number of samples, no specific time point for the cfHb difference was found during the post-hoc analysis. The cfHb clearance decreased from 30.0 mL/min (20.0–36.4 mL/min) at 0.2 L processed cfHb-solution to 11.1 mL/min (0.0–20.0 mL/min) at 3 L to 0.0 ml/min (0.0–0.0 mL/min) at 24 liters processed cfHb solution (Fig 7).

### Control setup

A third setup using the circular setup but without a Cytosorb^®^ adsorber was performed for 24 hours to ensure the stability of the cfHb solution. A volume of 2.1 L of the cfHb solution was used. The concentration of cfHb in the cfHb solution remained unchanged at 0.6 mmol/L within 24 hours.

## Discussion

In the present *in vitro* pilot study, the capability and capacity of the Cytosorb^®^ adsorber to reduce cfHb from canine cfHb solution were examined in two different setups. A median cfHb reduction of 17.8% and 41.3% was achieved in the linear and circular setups, respectively. The Cytosorb^®^ adsorber appears suitable for cfHb reduction from canine cfHb solution.

The hemoglobin molecule is primarily hydrophilic, with some hydrophobic elements. The hydrophobic parts are especially responsible for oxygen binding. The hemoglobin’s molecular weight of 64.5 kilodaltons is in the upper range of the adsorption spectrum of the Cytosorb^®^ adsorber [47]. Thus, hemoglobin barely meets the requirements to be adsorbed by the Cytosorb^®^ adsorber. The effect of the Cytosorb^®^ adsorber of cfHb has been demonstrated in humans. One study in humans during prolonged cardiac surgery has proven a reduction of the cfHb with the Cytosorb^®^ adsorber [1]. Another case series reported an effective reduction of cfHb in humans undergoing ECLS with the Cytosorb^®^ adsorber over 24 hours and 4 days [44]. The findings indicate that the Cytosorb^®^ hemoperfusion can effectively reduce cfHb concentrations in human patients.

In the linear setup of the present study, the strongest post-adsorber cfHb concentration changes occurred during the first liter of processed cfHb solution. Only minor changes in cfHb were observed after processing the first liter of cfHb solution. The cfHb clearance of 47.8 mL/min (40.0–52.9 mL/min) at 0.1 L of processed cfHb-solution decreased quickly after 0.4 L of processed cfHb-solution. This phenomenon can be attributed to the increasing concentration of cfHb within the Cytosorb^®^ adsorber, resulting in a diminished concentration gradient between the cfHb solution and the adsorber. Consequently, although the cfHb concentration before the adsorber remained consistent, the adsorption rate gradually declined as the experiment progressed. The concentration of cfHb within the Cytosorb^®^ adsorber increased as more cfHb solution was processed, further reducing the concentration gradient. Hence, despite the stable pre-adsorber concentration, the amount of cfHb adsorbed varied throughout the process. In the first test run of the linear setup, a higher starting concentration of 2.3 mmol/L was used compared to the other test runs with starting concentrations of 1.7 and 1.5 mmol/L. In the first test run, a significantly higher amount of cfHb was adsorbed compared to the other two runs. This suggests that higher starting concentrations and thus a higher concentration gradient of cfHb may lead to a higher adsorption rate. However, there is currently a lack of comparative studies employing a similar linear setup to substantiate these hypotheses.

The initial sharp decrease in cfHb concentrations suggests a short-term high efficacy of this method. However, the subsequent decrease of the cfHb clearance during Cytosorb^®^ hemoperfusion highlights the necessity of periodic adsorber replacement to maintain low cfHb levels over a longer treatment duration, which was already presented in a case report in humans [44].

In the circular setup of the present study, the strongest decrease in cfHb concentration was observed within the first 2 liters. The circular setup achieved a greater and more sustained cfHb reduction during processing of 13 L of cfHb solution, before the stabilization of cfHb concentrations before and after the adsorber occurred. This suggests a saturation of the Cytosorb^®^ adsorber in patients with smaller blood volumes without ongoing hemolysis. The steady decrease followed by stabilization indicates that adsorption efficiency is closely linked to the concentration gradient between cfHb and the adsorber, which diminishes over time. Unlike the linear setup, which was limited by rapid saturation of the adsorber, the circular setup benefited from continuous recirculation, mimicking more realistic *in vivo* conditions.

In humans undergoing ECLS over 24 hours up to four days, the cfHb concentration decreased within the first 12 hours but increased again thereafter. Therefore, a new Cytosorb^®^ adsorber was integrated into the setup after 24, 48, 72, and 96 hours. After 36 hours, another hemoglobin increase was recorded. After that, a continuous cfHb decrease, with only a mild additional peak after 60 hours, was observed. The lowest cfHb concentration was reported after 84 hours [44].

Results of the circular setup in the present study, conducted over an extended duration, suggest that time also influences the adsorption process. A previous report in humans indicates that the Cytosorb^®^ adsorber, integrated into an ECLS setup, maintains an effective adsorption of cfHb for up to 24 hours [44]. This prolonged operation duration could account for the diminished adsorption rate observed in the linear setup, since it was run for a shorter period compared to the circular setup. Meanwhile, the circular setup was continued until the concentration of cfHb stabilized before and after the Cytosorb^®^ adsorber, and no changes were observed for 10 L. Initially, a rapid decline in cfHb concentration was evident in the circular setup until stabilization occurred. After 13 L of processed cfHb solution, no further cfHb reduction occurred. In this configuration, the adsorption rate diminished in parallel with the decreasing concentration of cfHb, reflecting the diminishing concentration gradient between the cfHb solution and the adsorber. Once the cfHb concentration before and after the Cytosorb^®^ adsorber was stable, no further reduction occurred. Similar phenomena have been observed in studies involving lipopolysaccharides (LPS), interleukins, and tumor necrosis factor (TNF), as well as in human trials using cfHb in which concentrations declined rapidly initially before leveling off over time [31,44,48]. Additionally, *in vivo* trials, administering LPS to stimulate the immune response, revealed effective elimination of interleukins and TNF by the Cytosorb^®^ adsorber, with concentrations aligning before and after the adsorber over time [31,44,48]. Also, a degradation of the adsorber beads or clogging of the adsorption material could be a reason for the lower adsorption rate [49].

The key distinctions between the setups were that the linear setup exhibited a rapid but limited cfHb reduction, reaching a plateau after 2.2 L of processed cfHb solution, demonstrating the adsorbers’ saturation. The linear setup, designed to mimic conditions of ongoing hemolysis, showed an initial rapid reduction in cfHb concentration but quickly reached saturation, with no further removal after processing 2.2 L. This suggests that for applications where cfHb levels are consistently high, periodic replacement of the adsorber may be required to maintain efficacy. The rapid saturation in the linear setup also highlights the impact of high initial cfHb concentrations, where the adsorption rate declines as the concentration gradient diminishes. The circular setup’s extended adsorption capacity suggests that continuous recirculation may be a more effective strategy for sustained cfHb removal.

In the control setup, the concentration of cfHb remained stable at 0.6 mmol/L for 24 hours. This leads to the conclusion that cfHb is stable without relevant self-degradation over a long period (24 hours) and demonstrates that the decrease in cfHb concentration is caused by the adsorber.

The flow rate of the cfHb solution used in the present pilot study was 100 mL/min. This is the minimal flow rate recommended by the Cytosorb^®^ manufacturer. The flow rate might have influenced the adsorption efficiency in different ways. A higher flow rate leads to a decreased contact time between the cfHb solution and the adsorber, which could reduce the adsorbed cfHb per liter. On the other hand, a higher blood flow increases the processed cfHb solution volume.

Saturation of the adsorber can also be achieved by substances other than cfHb, such as bilirubin, myoglobin and cytokines (24). Given the analogous composition of the initial components, it is unlikely that a greater adsorption of other substances occurred in the second and third runs of the linear setup. If adsorption of other substances occurred, a similar adsorption is assumed during all test runs. A higher cfHb starting concentration and therefore a higher concentration gradient are more likely to be the reason for the differences between the test runs.

Anticoagulation was achieved with 10,000 I.U./L of heparin. This is a 10-fold dose that would be administered to a clinical patient treated with extracorporeal therapies. The Cytosorb^®^ adsorber binds different anticoagulants, such as ticagrelor or rivaroxaban (25, 26). The potential adsorption of heparin by the Cytosorb^®^ adsorber is an important consideration, particularly in an *in vitro* setting where anticoagulation relies solely on external supplementation and no metabolization of heparin occurs. Although the adsorber is primarily designed to bind hydrophobic substances and is highly polar, its partial adsorption cannot be fully excluded. While the manufacturer does not list heparin as a common target molecule, clinical observations suggest that anticoagulation may be affected during extended Cytosorb^®^ use [50]. Recent studies have shown a significant adsorption of the anticoagulant Argatroban by the device [51]. In the present study, a high heparin concentration (10,000 IU/L) was used to ensure adequate anticoagulation. However, as the effect of heparin was not controlled, adsorption of heparin and clotting of the cfHb solution remain possible influencing factors, despite clotting was not observed in the tubing system.

Currently, no veterinary study evaluates the effect of the Cytosorb^®^ adsorber on cfHb. Two publications report the use of the Cytosorb^®^ adsorber in veterinary patients. The first case series describes the use of the Cytosorb^®^ adsorber in three dogs with immune-mediated hemolytic anemia (IMHA) receiving a combination of therapeutic plasma exchange (TPE) and hemoperfusion. The Cytosorb^®^ adsorber could be a beneficial additional tool to control severe cases of IMHA [45]. Another case report used the Cytosorb^®^ adsorber in combination with immunoadsorption and hemodialysis in a dog with Leishmaniosis-induced Glomerulopathy to reduce cytokines and light chain proteins [52].

The present study has some limitations. One is the low number of test runs. Given the most likely stable adsorption capacity of the adsorber, the authors assume the number of *in vitro* runs is sufficient to estimate the adsorber capacity. Further prospective *in vivo* studies are required to assess the adsorption capacity *in vitro* with larger numbers of runs and larger cohorts of different cfHb concentrations, as well as in clinical patients. Furthermore, the time point 0 represents the situation when the first milliL of the cfHb solution had passed the sampling port. A dilution with residual priming solution might have led to false low cfHb concentrations during the first 2 sampling time points. The priming solution of a total of 200 ml was also mixed into the hemolyzed blood solution, which decreased the end concentration of the cfHb in the waste bag in the linear setup and in the reservoir bag in the circular setup. This represented about 10% of the volume of the cfHb solution in the circular setup and about 6% of the volume of the linear setup and contributed partially to the decrease of the cfHb concentrations.

The starting concentration of cfHb and the amount of the cfHb solution varied. This was caused by the different volumes and concentrations of the blood bags used to create the cfHb solution and decreases the comparability of the test runs more difficult. Additional studies with standardized blood volume and cfHb concentration would be beneficial for more accurate comparison. As a one-compartment model was used, the distribution dynamics of cfHb, as well as ongoing hemolysis in patients, cannot be addressed *in vitro.*

It is unclear if the presence of red blood cells, platelets and cytokines in the plasma influences the efficiency of the Cytosorb^®^ adsorber. Nevertheless, the Cytosorb^®^ adsorber does not affect the albumin concentration or platelet count *in vivo* [53–55]. As white and red blood cells are not adsorbed by the Cytosorb^®^ adsorber, they most likely do not influence its efficiency [34]. On the other hand, the cfHb solution should not contain red or white blood cells. It cannot be excluded that cell detritus of the hemolyzed cells may influence adsorption capacity due to clogging of the adsorber.

This pilot study shows the potential of the Cytosorb^®^ adsorber to reduce the cfHb concentration from canine blood The Cytosorb^®^ hemoperfusion for hemoglobin reduction could be clinically used in patients with intravascular hemolysis, due to immune-mediated hemolytic anemia or Babesiosis. Future studies should focus on *in vivo* applications, particularly assessing the effect of blood flow rates, ongoing hemolysis, and adsorption saturation on performance in clinical settings. Further investigation into optimizing flow rates and adsorber replacement intervals may provide insights into maximizing cfHb removal in veterinary and human medicine. ^1, 42^

## Conclusions

Hemoperfusion using the Cytosorb^®^ adsorber effectively reduced cfHb from a canine cfHb solution *in vitro.* Especially in the circular setup, cfHb-clearance decreased continuously. No further reduction in cfHb was observed after processing 13 L of cfHb solution. The circular setup provided superior and sustained clearance compared to the linear setup, with adsorption ceasing only after 13 L due to adsorber saturation. These results support the potential clinical utility of Cytosorb^®^ in managing hemolytic diseases such as IMHA or Babesiosis in veterinary medicine. However, additional studies are needed to evaluate the *in vivo* efficacy of hemoperfusion using the Cytosorb^®^ adsorber.
